# Faecal microbial biomarkers in early diagnosis of colorectal cancer

**DOI:** 10.1111/jcmm.17010

**Published:** 2021-11-09

**Authors:** Chinasa Valerie Olovo, Xinxiang Huang, Xueming Zheng, Min Xu

**Affiliations:** ^1^ Department of Biochemistry and Molecular Biology School of Medicine Jiangsu University Zhenjiang China; ^2^ Department of Microbiology Faculty of Biological Sciences University of Nigeria Nsukka Nigeria; ^3^ Department of Gastroenterology Affiliated Hospital of Jiangsu University Zhenjiang China

**Keywords:** adenoma, carcinoma, colorectal cancer, dysbiosis, early diagnosis, faecal microbiota

## Abstract

Colorectal cancer (CRC) is ranked as the second most common cause of cancer deaths and the third most common cancer globally. It has been described as a ‘silent disease’ which is often easily treatable if detected early—before progression to carcinoma. Colonoscopy, which is the gold standard for diagnosis is not only expensive but is also an invasive diagnostic procedure, thus, effective and non‐invasive diagnostic methods are urgently needed. Unfortunately, the current methods are not sensitive and specific enough in detecting adenomas and early colorectal neoplasia, hampering treatment and consequently, survival rates. Studies have shown that imbalances in such a relationship which renders the gut microbiota in a dysbiotic state are implicated in the development of adenomas ultimately resulting in CRC. The differences found in the makeup and diversity of the gut microbiota of healthy individuals relative to CRC patients have in recent times gained attention as potential biomarkers in early non‐invasive diagnosis of CRC, with promising sensitivity, specificity and even cost‐effectiveness. This review summarizes recent studies in the application of these microbiota biomarkers in early CRC diagnosis, limitations encountered in the area of the faecal microbiota studies as biomarkers for CRC, and future research exploits that address these limitations.

## 1 INTRODUCTION

Colorectal cancer (CRC) is the second most common cancer in women and the third in men.^1^, ^2^, ^3^, ^4^ It ranked second in mortality and third in incidence among cancers worldwide in 2020.^5^, ^6^, ^7^ Even though cases in some other countries are on the rise, most CRC cases occur in Western countries with an annual increase in incidence rates.

Colorectal cancer has been described as a ‘silent disease’ which develops after many years following a stepwise series of genetic changes termed the adenoma‐carcinoma sequence.^8^ This cancer occurs from complex interactions between genetic/epigenetic, environmental and lifestyle factors. The predictable sequence with which CRC takes to develop makes screening very paramount in the fight against this particular cancer. Not only is the sequence predictable, but it also takes years, about 10–15, to fully develop (though it is faster occurring in Lynch syndrome), that is, the adenoma‐carcinoma (polyp to cancer) sequence. This, therefore, means that a window of opportunity for early diagnosis occurs for the disease since symptoms appear in the late stages of the disease. It is therefore important that with the aid of accepted biomarkers, early diagnostic approaches are instituted as this greatly impacts survival rate.^9^, ^10^

O’Connell and his colleagues stated that the early diagnosis of CRC at localized stages (American Joint Committee on Cancer (AJCC) stages 0, I or II) increases the survival rate to >80% but if diagnosed in the late stages, when cancer has metastasized, (AJCC stage III or IV), the survival rate is decreased to <10%.^11^ A later documentation also stated that the survival rate of individuals who are diagnosed early or have localized CRC is approximately 90%, but the rate drastically reduces to about 14% in patients with metastasized CRC.^12^, ^13^ This is the basis for the recommendation of population wide‐screening and prevention programs in several countries.

Screening is done primarily to detect cancer at an early treatable stage,^14^ thereby preventing carcinogenesis through adenoma detection and complete excision. A review study by Nguyen & Weinberg also emphasized that as a result of the long process involved in the adenoma‐carcinoma progression of CRC, detection of CRC early enough positively impacts survival rates as the adenoma can be excised thus, preventing its progression to carcinoma and patients could be identified even before symptoms begin to manifest.^15^

The major aim of any CRC diagnostic method is to reduce the overdependence on colonoscopy, the standard for CRC diagnosis, which poses greater risk and is rather very expensive.^15^, ^16^ Besides the fact that colonoscopy presents a reasonable level of discomfort for the patient, it is also invasive, relatively costly and poses some health risks such as post polypectomy, puncture of the colon, intraperitoneal bleeding and the possibility of infection.^7^, ^9^, ^15^, ^17^, ^18^ Thus, the need for other detection strategies that are both non‐invasive and highly effective. Screening methods with high specificity and sensitivity for adenoma detection will by far enhance chances at detecting curable tumours, thus grossly decreasing the mortality and morbidity rates associated with CRC.^9^

Stool tests remain the focal point for non‐invasive options in CRC diagnosis.^9^ Non‐invasive stool‐based methods such as the guaiac faecal occult blood test gFOBT and faecal immunochemical test FIT are however not reliable in the detection of adenomas although FIT has now largely supplanted gFOBT due to its improved sensitivity. Additionally, some basic limitations such as high cost and reduced sensitivity in adenoma detection exist in the multitarget stool DNA test which has been reported to possess improved diagnostic accuracy compared to FIT and was approved by the US Food and Drug Administration in 2014^15^ for the screening of asymptomatic average‐risk CRC persons.^19^ Hence, an urgent need for new, non‐invasive CRC screening methods with increased sensitivity and specificity, for the detection of adenomas and early‐stage CRCs.^13^

A biological entity that can be employed to determine the presence or the progression of any particular disease or measure the effects exerted by the treatment given is known as a biomarker. Several important features make for a good biomarker. Such qualities are high specificity, safety, sensitivity, easy to use as a determinant and practical for ascertaining accurate diagnosis as well as enabling appropriate treatment options.^20^ More lives will undoubtedly be saved if biomarkers with the above‐mentioned qualities in addition to being cost‐effective are discovered and employed in the management of CRC. Thus, in essence, many Scientists are doggedly researching this biomarker issue for CRC diagnosis as it not only guarantees early detection of CRC but will also aid in the development of personalized and targeted treatment for affected individuals.

Certain factors such as diet, age, disease, antibiotics, stress, mode of birth, host genetics and other environmental factors determine the composition and diversity of the gut microbiota while the gut microbiota in turn, influences the health of the host with an effect on genes, proteins and metabolites production.^21^, ^22^ In a state of homoeostasis, the gut microbiota is beneficial to the host; however, a dysbiotic gut‐state can arise if any perturbations occur in the balance of the gut microbiota. Dysbiosis, a metabolic condition that results from the imbalance in a host's gut microbiota, may result in disruption of the host metabolism and immune function leading to several diseases, including inflammatory bowel disease, autoimmune diseases, diabetes, obesity, irritable bowel syndrome, cardiovascular diseases and even cancer.^21^, ^22^, ^23^, ^24^, ^25^, ^26^, ^27^

Many gastrointestinal cancers such as pancreatic,^28^ liver,^29^ gastric^30^ and colorectal cancers^31^ have been implicated and various researchers have reported the major role played by dysbiosis in the development of adenoma and CRC.^32^, ^33^, ^34^, ^35^ With the aid of high‐throughput environmental sequencing techniques, a comprehensive makeup of the microbial ecosystem in healthy and diseased states has been made possible.^36^, ^37^ It is in recent years that microbes colonizing the gut environment were implicated as potential biomarkers for CRC screening. *Fusobacterium nucleatum*, *Solobacterium moorei*, *Peptostreptococcus stomatis* and *Parvimonas micra* which are majorly associated with host‐gut microbiome imbalance that occurs in CRC have been demonstrated by several studies as potential biomarkers for early CRC screening.^38^, ^39^, ^40^, ^41^

**FIGURE 1:**
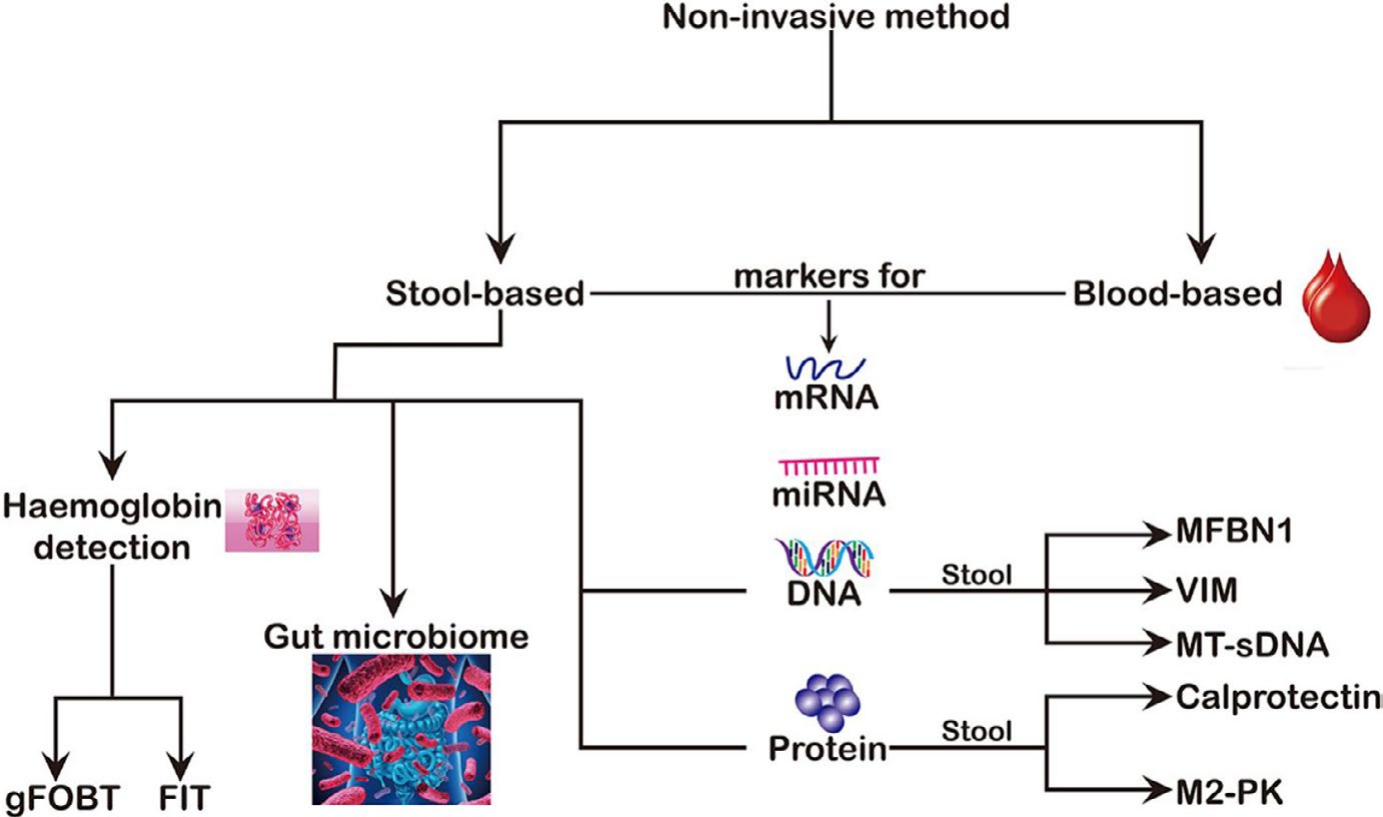
Current Non‐invasive Stool‐based Methods for Early Colorectal Cancer Detection. The two main methods, blood‐based and stool‐based are centred on the same biomarkers. Stool‐based biomarkers, which are our focus, have been clearly represented in the diagram. FIT, faecal immunochemical test; gFOBT, guaiac faecal occult‐based test; M2‐PK, tumour M2 pyruvate kinase; MFBN1, methylated fibrillin‐1; MT‐sDNA, multitarget stool DNA test; VIM, vimentin

In this review, we discuss the recent updates on the application of faecal microbiota biomarkers in the early diagnosis of CRC and it also highlights current findings in gut microbiota metabolites as biomarkers for early CRC detection. Furthermore, the challenges facing this research area and possible future research questions needing urgent answers that address these challenges were explored. The two main non‐invasive methods for the diagnosis of CRC is illustrated in Figure 1 with emphasis on the stool‐based biomarkers.

## 2 GUT MICROBIOME AND COLORECTAL CANCER

The anaerobic bacteria makeup of a healthy adult even with subtle disparities that exist in different individuals among various populations are made up of two major key phyla (comprising >90% of all the microbiome bacteria population); the Firmicutes and the Bacteroidetes.^42^

These intestinal microorganisms, particularly, the bacteria which are the most studied are primarily involved in modulating host metabolism such as synthesis of certain essential vitamins like K and B, extraction of energy from indigestible carbohydrates, protection of the gut from colonization by enteric pathogenic organisms, and immune system modulation.^43^, ^44^ The gut, in turn, provides these organisms with an adequate environment, abundant in nutrients, with the right range of pH and oxygen concentration necessary for their growth and metabolism.^45^

An interesting fact is that compared to the level of bacteria in the small intestine, the bacteria levels in the large intestine are estimated to be 12‐fold higher and more cancers are found to occur in the colon than in the small intestine. This suggests a possible connection between colorectal carcinogenesis and the microbiome (majorly bacteria) of the gut.^46^, ^47^

Several studies have suggested that the complexity of CRC disease stems from the interaction of several factors which result in the disease. Genetic, epigenetic,^15^, ^48^ and environmental factors have been implicated. The most important environmental factors that influence CRC have been identified as lifestyle and diet,^49^ and dietary patterns are known to affect the gut microbiota in real time.^50^

Studies have linked the gut microbiota in the development of intestinal adenoma which could progress to CRC.^51^ Conversely, healthy gut microbiota is closely associated with a reduced risk of advanced adenoma.^52^, ^53^ There are several proofs that many known risk factors for CRC are chiefly implicated in the structure and function of the gut microbiota.^54^ These in turn influence immune responses, host metabolism, changes in cancer‐driving genomics and epigenomics, and ultimately, CRC development. For instance, just as increased intake of dietary fibres has been shown to result in the enrichment of such bacteria like *Bifidobacterium* and *Lactobacillus* spp., by causing increased production of the beneficial short‐chain fatty acids (SCFAs) in the gut via fermentation of these dietary fibres by the bacteria,^8^ so also is the high consumption of diets rich in red and processed meat known to be unhealthy. This is as a result of the high sulphur‐containing amino acids and inorganic sulphur present in them which leads to an abundance of sulphidogenic bacteria for metabolizing these proteinous foods, yielding such metabolites as hydrogen sulphide, ammonia or polyamines known to produce genotoxic components in the gut. As a consequence, DNA damage in intestinal epithelial cells results in fostering colonic carcinogenesis.^55^, ^56^

The gut flora of patients with CRC has been found to be composite of certain bacterial species such as *Fusobacterium*, *Solobacterium*, *Bacteroides*, *Peptostreptococcus* and *Parvimonas*.^38^ It is such that the diversity and composition of their gut microbiota are significantly different from their healthy counterparts. Studies have shown that these microbial compositions can be detected across different ethnic groups and races. Furthermore, since some of these microbial genes are enriched in early‐stage CRC,^38^ there exists the potential for their application as biomarkers in early CRC detection.

Because the colonic mucosa is in close contact with the gut microbiota and its metabolites, there is bound to be stimulation of immune responses by these bacteria and this may subject the mucosa to continuous low‐grade inflammation and chronic inflammation is an established risk factor for several cancers including CRC.^8^ Various other researches have also linked chronic inflammation to various cancer types.^45^, ^57^, ^58^

It should be noted that under healthy physiological conditions, the gut bacteria and the host are in a state of homeostasis. However, when the balance of the gut microbiome is altered by certain factors such as alcohol intake, some diet types, or even antibiotics treatment, serious problems could arise.^43^ This state of the gut known as dysbiosis has been implicated as a major probable cause of certain diseases such as diabetes type 2, cardiovascular diseases, inflammatory bowel disease and CRC.^50^, ^59^

Dysbiosis, a condition that arises from the imbalance of the gut microbiota, has been connected by several researchers to the development of adenomas and CRC.^32^, ^33^, ^60^, ^61^, ^62^ Even though the mechanism by which dysbiosis could result in CRC is yet to be fully understood, chronic inflammation is believed to be a major factor. Concurrent with the metabolic shift in the microbiome of CRC patients, Zeller and colleagues, reported an increased array of proinflammatory and pathogenicity processes resulting from the presence of many Gram‐negative bacteria. As a result, inflammation‐induced carcinogenesis ensues due to the lipopolysaccharides present on the outer membrane of these bacteria since they elicit an inflammatory signalling cascade by binding to Toll‐like receptor 4 in the epithelial cell^63^, further confirming the tumour‐promoting activity of inflammation.

One of the earliest large series studies that showcase this, is the research by Sobhani and colleagues. The study showed the relationship between the microbiota of a healthy human and when in a diseased state. Differences between the gut microbiota of normal individuals and CRC patients were observed. Pyrosequencing was done after 16S rRNA PCR followed by principal component analysis (PCA) using the stool bacterial DNA from patients with CRC and those that have a normal colonoscopy. With this, the researchers were able to determine that more *Bacteroides* and *Prevotella* spp. were represented in individuals with cancer compared to the normal group. Furthermore, they found that IL‐17 immune cells were significantly expressed in the mucosa of the cancer patients than in those that have normal colonoscopy indicating that the composition of the gut microbiota could possibly impact the mucosal immune response.^64^ In another study by Nakatsu and colleagues, the association of dysbiosis with CRC was investigated by characterizing the mucosal microorganisms of the gut associated with different stages of colorectal carcinogenesis. Using paired samples of adenoma and adenoma‐adjacent mucosae, carcinoma and carcinoma‐adjacent mucosae, and healthy controls, they determined that correlations of certain bacterial taxa in adenoma, indicate early signs of dysbiosis and in carcinoma, more co‐exclusive relationships are found. They concluded that a well‐defined taxonomy of microbial consortium is associated with the occurrence of CRC.^65^ Among other observations made by Sun and colleagues in their study using their in‐house generated CRC mouse model, an increase in the bacteria inflammatory groups, Bacteriodetes and Porphyromonadaceae led to their proposition that both the interactions of bacteria in a dysbiotic gut and the effects of the metabolites generated on interrelated molecular events contribute to the advancement of colorectal carcinogenesis.^66^

Yet, observations made in another study carried out using Apc^min/+^ mice was that the mice group administered with faeces from CRC patients had more intestinal tumours in comparison with those fed with phosphate‐buffered saline PBS or faeces from healthy controls. They further observed that along with increased tumour proliferation, there was decreased tumour cells apoptosis, gut barrier function failure and upregulation of proinflammatory cytokines. Also, an increased abundance of pathogenic bacteria was seen after the feeding with the faeces of CRC patients. This altered gut microbiota not only induced low‐grade inflammation but also promoted the progression of intestinal adenoma in Apc^min/+^ mice.^32^

**FIGURE 2:**
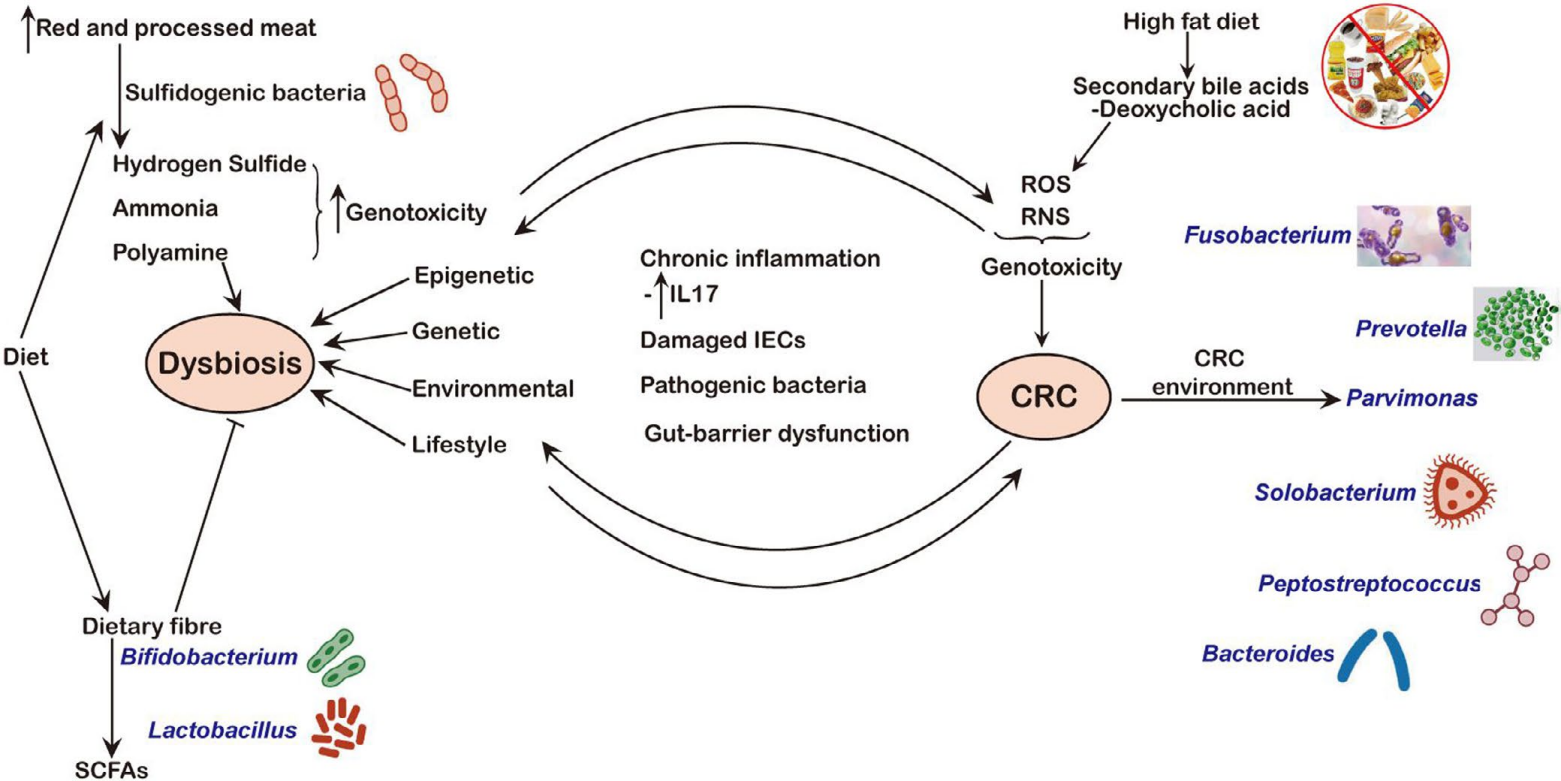
Link between Dysbiosis and CRC. In addition to the similarity in factors that trigger the onset of both dysbiosis and CRC like a diet that produces genotoxic metabolomics, the two conditions also share many common damages associated microenvironmental factors. While it is not certain whether dysbiosis is the cause or consequence of CRC, it is well established that CRC is characterized by the unique composition of the gut microbiome that can sometimes serve as a metagenomics biomarker. IECs, intestinal epithelial cells; IL–17, interleukin 17; RNS, reactive nitrogen species; ROS, reactive oxygen species; SCFAs, short‐chain fatty acids

All of these point to the close link that exists between dysbiosis and CRC. However, a conclusion is yet to be reached on whether dysbiosis is a cause or consequence of CRC disease.^51^, ^67^, ^68^ The analysis of the microbial composite of faeces could serve as a predictive factor for the risk of developing CRC, because these changes occur even in adenoma stages, hence, potential for their use in early diagnosis of the disease. This relationship between dysbiosis and CRC is illustrated in Figure 2 below.

## 3 FAECAL BIOMARKERS IN EARLY COLORECTAL CANCER DIAGNOSIS

### 3.1 *Faecal microbiota*/*metagenomics*

Variations in the gut microbiota composition and diversity between individuals diagnosed with adenoma or CRC and healthy individuals using faecal samples emphasize the potential of faecal microbiome usage in early, non‐invasive diagnosis of CRC. Different approaches were employed by various researchers in the study of the gut microbiome signatures in CRC. Methods ranging from amplification and sequencing of various variable regions (V1, V2, V4) of the 16S rRNA of the extracted stool DNA,^40^, ^63^, ^69^ to shot‐gun metagenome sequencing of the faecal samples.^41^, ^63^, ^70^ qPCR was also used in some studies to quantify the abundances of the target microbial gene markers^41^, ^70^, ^71^ present in the samples of interest relative to the selected controls.

Zeller and research team in their study identified *Fusobacterium* species (*Fusobacterium nuleatum* subspp. *vicentii* and *Fusobacterium nucleatum* subspp. *animalis*), *Peptostreptococcus stomatis* and *Porphyromonas asaccharolytica* as the most abundant and discriminative species enriched in the faecal samples of CRC patients relative to the controls. These microbial markers also correlated with the progression of CRC from early to late and metastasizing stages of cancer. A robust enrichment in the early‐stage CRC patients was particularly apparent for *Peptostreptococcus stomatis* and the two species of *Fusobacterium* compared to the controls.^63^ Zackular and colleagues combined the microbiome data obtained from the characterization of faecal samples of healthy individuals, adenoma and carcinoma patients’ groups with recognized clinical CRC risk factors such as age, race and body mass index. This significantly enhanced diagnostic distinction in healthy, adenoma and carcinoma subjects relative to risk factors alone.^69^ Yu and fellow researchers in their study identified 20 microbial gene markers that distinguished CRC and control microbiomes. They also validated 4 of the markers (two transposases from *Peptostreptococcus anaerobius*, m1704941, butyryl‐CoA dehydrogenase from *Fusobacterium nucleatum* and *rpo*B gene encoding RNA polymerase subunit β *from Parvimonas micra*) in Danish, French and Austrian cohorts. qPCR measurements of two of these genes classified correctly, patients with CRC in an independent Chinese cohort. These genes were shown to be enriched in the patients’ microbiome in the early stages (I – II) of CRC emphasizing the potential for their application in the early diagnosis of CRC.^38^

These studies portend that some particular bacteria genera such as *Fusobacterium* and *Peptostreptococcus*, are almost always present in a CRC‐associated dysbiotic gut. The accumulation of *Fusobacterium* even in the early stages of CRC in some patients^72^ further portrays the possibility of their application (though in combination with other specific microbial markers) in the early diagnosis of the disease. Additionally, many of the microbial markers identified were validated in independent cohorts of other foreign nationals (either by direct sampling or the use of previously published data). This validation mostly proved effective in distinguishing adenoma and early‐stage CRC patients from healthy individuals.

The ratio of *F. nucleatum Fn* to some important probiotics in the gut has also been projected as a useful biomarker for the early diagnosis of CRC. Guo and colleagues reported that the microbial ratio of *Fn* to *Bb Bifidobacterium* (*Fn*/*Bb*) in their study had high sensitivity and specificity of 84.6% and 92.3%, respectively, in the detection of CRC while the combination of *Fn*/*Bb* and *Fn*/*Fp Fecalibacerium prausnitzii* gave a sensitivity of 90.0% and a reduced specificity of 60.0% in the detection of stage 1 (early) CRC. Moreover, *Fn* correlated inversely with *Fp* in the CRC group and this relationship was significant when compared to the control groups indicating a CRC‐associated dysbiosis.^71^

The relative abundance of *Clostridium symbiosum* was measured by qPCR in colorectal adenoma CRA patients, early/advanced CRC patients and healthy controls, and prediction accuracy compared to *F. nucleatum*, FIT, and carcinoembryogenic antigen CEA (blood‐based biomarker).^73^ *C. symbiosum* was found to perform better than all the other biomarkers employed in the study as a significant stepwise increase of the organism's abundance level was observed in CRA, early CRC and advanced CRC patients compared to the healthy controls.^73^ This report makes *C. symbiosum* a prominent promising biomarker for early and non‐invasive CRC diagnosis.

Improved diagnostic performance for discriminating AP (adenomatous polyps) and CRC patients from the normal control group was observed in a five bacteria panel which included *F. nucleatum*, *Enterococcus fecalis*, *Streptococcus bovis*, Enterotoxigenic *Bacteroides fragilis* (ETBF) and *Porphyromonas* spp. With this bacteria panel, the AUROC increased to 0.97 and a sensitivity of 91.4%, and specificity of 93.5% was obtained using the simple linear combination model.^51^ In the case of CRC, a statistically significant increasing range of *F. nucleatum*, *E. fecalis*, *Porphyromonas* spp. and *P. gingivalis* biomarkers distinguished early‐stage CRC patients, from AP patients and healthy controls individuals.^51^

A significantly higher level of *Parvimonas micra* was found in the faecal samples of CRC patients compared to the normal controls and it was applied alone for distinguishing early‐stage CRC patients from the controls. The sensitivity and specificity obtained were 60.5% and 87.3%, respectively, and the combination of the bacterium with other microbial biomarkers (*F. nucleatum* and colibactin toxin‐producing *clb* A + bacteria) improved the sensitivity. The study further proposes that in combination with these microbial faecal biomarkers, identification of patients bearing ‘high risk’ microbial patterns indicative of increased cancer risk may be possible with *P. micra*.^18^

Zagato et al. (2020)^74^ in their study identified two gut microorganisms; *Faecalibaculum rodentium* and its human homologue, *Holdemanella biformis* that were not only anti‐tumorigenic but also had a great reduction in their composition during colorectal carcinogenesis. Interestingly, as early as 8 weeks (when the tumour growth had started), genera differences were observed between the Apc^Min/+^ mice and the C57BL/6 wild‐type WT littermates which served as the control. The paired‐end reads gave a quantitative reduction of *F. rodentium* which even became more prominent at 12 weeks. In fact, only the reads credited to *F. rodentium* (among the ten taxonomic units most abundantly represented in the WT mice) were found to be strongly under‐represented in the Apc^Min/+^ mice compared to the WT mice. By interrogating a data set of shot‐gun microbiome analysis performed in patients bearing advanced colorectal adenomas and other *in vitro* and *in vivo* experiments employing CRC cell lines, tumour tissues from CRC patients and mice, they were able to identify human *H. biformis* as very closely related to the mouse *F. rodentium* and could be referred to as a human homologue of the bacterium. In essence, the possibility of translating these research findings in the early detection of CRC at the adenoma stage exists.^74^

Clos‐Garcia and co. integrated data obtained from metabolomics and metagenomics in their study. They reported that the genera *Fusobacterium*, *Parvimonas* and *Staphylococcus* were increased in CRC patients and became more abundant as the disease progressed.^40^ Lachnospiraceae family, on the other hand, were reduced (Table 1). These bacteria genera served to clearly distinguish the CRC group from adenoma and control groups. Interestingly, the genera *Adlercreutzia* was found to be more abundant in the faeces of adenoma patients compared to the control and carcinoma groups suggesting the possibility of its application as a biomarker in early CRC detection. Barely any differences were reported to exist between the microbiome of control and adenoma groups in all the analytical methods employed in the study.

**TABLE 1:** Some major findings on faecal microbial biomarkers in early detection of colorectal cancer.

Hypothesis	Method used	Biomarkers	AUC	Some major findings	Validation in other independent cohort study	References
Novel microbiome biomarkers + Known clinical risk factors for CRC improves diagnostic performance	16S	6 OTUs + Age, gender and race. **5 OTUs + age and ra**ce. 6 OTUs + age, race and BMI **4 OTUs + BMI**	0.936 **0.896** 0.922 **0.963**	Significant improvement in distinguishing healthy individuals from patients with colonic lesions (adenoma and carcinoma). **Gave great differentiation between adenoma and healthy groups**. Significantly improved the ability to distinguish between healthy individuals and carcinoma patients. **Provided the greatest differentiation between adenoma and carcinoma patients**.	NO	(55)
Given the unsatisfying sensitivities observed with FIT in the diagnosis of CRC and adenoma, stool‐based bacteria could serve as better non‐invasive diagnostic biomarkers of the disease.	Shotgun metagenomics + qPCR	Faecal *m3* from *Lachnoclostridium* spp. YL32 ***F*. *nucleatum***	0.675 **0.862**	*m3* performed better than *Fn* in differentiating adenoma patients from healthy controls while ***Fn* performed better than *m3* in CRC diagnosis**	YES	(56)
GM biomarkers in CRC screening differ based on region and highly accurate CRC screening could be achieved using GM biomarkers identified via comparison between the faecal microbiome of CRC patients with that of healthy family members.	Shotgun metagenomics + qPCR	22 microbial genes **A gene from *Coprobacillus* (belonging to the 22 microbial gene biomarkers above)**	See study	Significantly differentiates CRC cases from healthy families and biomarkers revealed regional tendency. **Distinct classification of CRC patients from healthy controls in an Independent cohort**.	YES	(27)
Possible identification of non‐invasive biomarkers from combined metabolomics and metagenomics data from faecal samples of controls and patients.	16S + UHPLC‐MS	*Adlercreutzia* (more abundant in Ad group) *Staphylococcus*, *Parvimonas*, *Streptococcus*, ˂Lachnospiraceae family in CRC group ***Fusobacterium*** ***Parvimonas*** ***Staphylococcus*** **˂Lachnospiraceae**	Ad vs CRC =0.870. Metabolites added =0.923. **CRC vs C = 0.887. Metabolites added =0.928**	The genera *Adlercreutzia* was found to be more abundant in the faeces of adenoma patients compared to the control and carcinoma groups. Increased relative abundance of *Staphylococcus*, *Parvimonas*, *Streptococcus* genera in CRC group. **Found to be more abundant in CRC patients relative to the control and adenoma groups while the Lachnospiraceae family decreased**.	NO	(26)
There is a possibility that an altered ratio of *Fn* to gut probiotics in stool of CRC patients associates with clinical outcome and might be a diagnostic biomarker for CRC	16S + qPCR	*Fn*/*Bb* ***Fn*/*Fp*** *Fn*/*Bb* +Fn/*Fp*	0.911 **0.914** 0.943	Had superior sensitivity (84.6%) and specificity (92.3%) in detecting CRC. **Showed excellent sensitivity (94.5%) and slightly lower specificity (71.28%) in distinguishing CRC from healthy controls**. Gave 60.0% specificity and 90.0% sensitivity in detecting stage I of CRC.	YES	(57)
Faecal microbiota could be employed in non‐invasive diagnosis of CRC irrespective of nationality	Shotgun metagenomics +16S	*Fusobacterium nuleatum* subspp. *vicentii* and *Fusobacterium nucleatum* subspp. *animalis*), *Peptostreptococcus stomatis* and *Porphyromonas asaccharolytica*	See study	All were enriched in the CRC patients and correlated with the progression of CRC from early to late and metastasizing stages of the cancer. A robust enrichment in the early‐stage CRC patients was particularly apparent for *P. stomatis* and the two species of *Fusobacterium* compared to the controls	YES	(49)
Associations of microbiome and CRC that are consistent across studies and less likely to be attributed to biological or technical confounders can be identified using meta‐analyses study based on shotgun metagenomics.	Shotgun metagenomics	29 species	See study	This study, through extensive validations firmly establishes globally generalizable, predictive taxonomic and functional microbiome CRC signatures (that are found even in early CRC stages) as a basis for future diagnostics	YES	(25)

Note

Abbreviations: Ad, Adenoma; AUC, Area under the receiver operating characteristic (ROC) curve; *Bb*, *Bifidobacterium*; C, Control; CRC, colorectal cancer; CRC, Colorectal cancer; *Fn*, *Fusobacterium nucleatum*; *Fp*, *Fecalibacterium prausnitzii*; GM, gut microbiome; OTUs, operational taxonomic units.

Researches have shown that *Adlercreutzia* is one of the most prominent bacteria recognized for its equol production from isoflavonoids present in the host's diet.^75^ Equol is implicated in the health of the host as it is known to be associated with higher levels of high‐density lipoprotein cholesterol and lower levels of dyslipidemia,^76^ thus, as reported by,^77^ has an indirect relationship with the risk of CRC. This could explain the trend observed in the study of^40^ since adenoma samples were more enriched in the *Adlercreutzia* bacteria compared to samples from CRC patients. Although the dietary habit of participants was not ascertained in the study, (as equol is produced from isoflavonoids in diets), the authors proposed that the changes seen in *Adlercreutzia* could be a result of the dietary patterns of the adenoma subjects. Future studies on this need to be carried out. Secondly, the effect of *Adlercreutzia* in adenoma patients can be studied since the equol it produces is associated with a lower risk of CRC. The possibility that higher levels may have a positive effect in CRC progression should be further investigated.

Metagenomics analysis study of faecal samples from CRC patients was shown to be significantly different from those of healthy live‐in family members. The 22 microbial genes identified in the study, which served as the screening biomarkers as well, showed high accuracy and sensitivity in differentiating CRC patients and healthy controls.^41^

The gene *m3* from *Lachnoclostridium* spp. has been identified by^70^ as a biomarker for early CRC diagnosis. There was a significant increase in the faecal *m3* and *Fn* from healthy controls, through the adenoma, to the carcinoma groups although faecal *m3* may be better than *Fn* in differentiating adenoma patients from controls. This is because at a specificity of 78.5%, sensitivities of 48.3% and 33.8% for adenoma were observed for *m3* and *Fn*, respectively.

Recent meta‐analysis studies of faecal metagenomics (using shot‐gun metagenome data) in the diagnosis of CRC have tried to validate the replicability of the microbiome biomarkers across various populations.^39^, ^78^ Wirbel and colleagues identified a set of 29 main species with significant enrichment in the CRC metagenomes. These identified CRC microbial signatures which were stated to be validated in diverse populations were also established to be present even in the early stages of CRC. This means that not only are they used for CRC detection, but also successful diagnosis of CRC in the early stages can be achieved irrespective of race and this would in essence, grossly reduce the mortality associated with the disease.^39^ Fascinatingly, disease‐specific signatures of a dysbiotic gut particular to CRC were applied in the study thus eliminating the possibility of including signatures from gut dysbiotic patterns resulting from other diseases such as inflammatory bowel disease and diabetes.^39^ Furthermore, Wu and colleagues employed Random Forest Classifier models in their meta‐analysis study to assess the CRC‐associated gut microbiome changes and the ability of the integrated features that distinguished the adenoma group from both the control and CRC groups. High diagnostic accuracy with AUC of 0.80 and 0.89 was achieved for the adenoma/control group and adenoma/CRC groups, respectively. These markers also showed high diagnostic accuracy in independent validation cohorts and were also ascertained to be adenoma‐specific.^79^ Table 1 below summarizes some major findings reported in studies on the use of faecal microbial biomarkers as non‐invasive tools in the early detection of CRC.

### 3.2 Metabolomics

Various studies have suggested that both the concerted activities of the gut microbiota and the influence of its metabolome contribute to the aetiology of CRC.^8^, ^69^ Thus, the possible application of these metabolites from a CRC‐influenced dysbiotic gut relative to that of healthy individuals as biomarkers for early CRC diagnosis emerged. The major approach used in the gut metabolome study in CRC patients is the ultra‐high performance liquid chromatography‐tandem mass spectrometry UHPLC‐MS technique^40^, ^80^ and proton nuclear magnetic resonance spectroscopy (^1^H NMR)‐based metabolomics approach.^81^, ^82^

SCFAs, particularly butyrate, have been reported to be anti‐carcinogenic due to their anti‐inflammatory properties^83^ (Figure 2). On the other hand, some gut bacteria have the ability of metabolizing primary bile acids into secondary bile acids which could promote CRC pathogenesis via the generation of reactive oxygen species (ROS) and reactive nitrogen species (RNS), known to be genotoxic (Figure 2). An example is deoxycholic acid and its effects have been captured in various review articles.^8^, ^83^

Recent studies have shown that an altered state of the gut microbiota results in the reduction of the concentration of the SCFAs.^84^ These SCFAs usually obtained from carbohydrate fermentation in the colon are known to be essential components needed for the maintenance of gut homeostasis. However, in a state of gut microbiota dysbiosis, fermentation of these carbohydrates yields a much lower concentration of the SCFAs than they would in healthy states. Their quantification in stools has been said to possibly function as biomarkers for non‐invasive diagnosis for various gut ailments. Thus, Niccolai and colleagues who proposed that the presence of AP and CRC disease could exhibit a specific faecal SCFAs’ characteristic, carried out a study to compare the concentration of SCFAs in faecal samples of AP and CRC patients and found that the total amount of SCFAs was significantly lower in CRC patients in comparison with the healthy controls. More so, the percentage composition of the faecal SCFAs was different in the healthy controls, compared to CRC and AP patients, and the healthy control groups could clearly be separated from the AP and CRC groups.^84^ This would suggest the potential application of faecal SCFAs determination as a biomarker in the early detection of adenomatous polyposis and CRC.^84^

Nevertheless, just as Wang and colleagues inferred that microbial metabolites may contribute to CRC development as they observed a reduction in butyrate‐producing bacteria in faeces of CRC patients from their study,^85^ and some studies also implying that there is an association between bacterial dysbiosis, metabolites and colorectal adenomas,^86^ more recent studies found that this association especially, between gut metabolites and colorectal adenoma or CRC is insignificant.^80^, ^87^ Sze and co. also reported in their 2019 study that there is a limited association between faecal SCFAs and colonic tumours. They determined whether a positive correlation existed between faecal SCFA concentration and the presence of colonic adenomas or carcinomas in a group of individuals with different stages of colonic tumours. However, after the measurement of the faecal concentrations of acetate, butyrate and propionate, there was no significant relationship between both the SCFAs concentration and tumour status of these individuals and their faecal microbiota composition and SCFAs concentration.^87^ Hence, no matter what method they implemented to associate any of these relationships that existed between faecal microbiota, SCFAs concentrations and tumour burden, it was still simply weak and could not serve as a predictive marker in the detection of adenomas or carcinomas in the colon. More so, Kim and co. investigated the faecal metabolomics profiles of patients with advanced adenoma and CRC. The control samples for the study were selected to match the age, race and sex distribution of the adenoma group. They reported that the concentrations of several classes of bioactive lipids such as sphingolipids, secondary bile acids and polyunsaturated fatty acids were higher in adenoma patients compared to the controls. Changes in most of these metabolites were consistent even in CRC patients but these observed changes in the identified bioactive lipids were not significant enough to be employed as metabolic biomarkers in the early diagnosis for CRC even when the metabolomics profiles analysis were paired with microbiota composition profiles. They nonetheless, served to provide better insights into the early events that occur in colorectal pathogenesis^80^ and further targeted experiments can provide deeper insights into CRC prevention strategies.

Kinross et al^88^ who sampled the gut mucosal microbiota using tumour tissues rather than faeces of patients reported that although 16S rRNA gene sequencing revealed that the ecology of the gut microbiota appears to be cancer‐stage specific and is strongly connected with features of poor prognosis. While fusobacteria and ε‐*Proteobacteria* were more abundant on tumour compared to adjacent normal mucosal tissue, and fusobacteria and β‐*Proteobacteria* levels increased as the cancer‐stage advanced. However, network analysis revealed that the bacteria associated with these poor prognostic features were not responsible for the alteration of the cancer mucosal metabonome. They further proposed that the mucosal microbiota in CRC only evolves with the cancer stage in order to meet the requirements of cancer metabolism and that from the ‘driver‐passenger model’ of Tjalsma and colleagues,^89^ these ‘passenger bacteria’ may be involved in the maintenance of cancer mucosal metabolic homeostasis but the metabolic functions may not be cancer‐stage specific. Thus, even though the 1H Magic Angle Spinning Nuclear Magnetic Resonance (MAS‐NMR) spectroscopy which was employed for metabonomic analysis exhibited an increased abundance of taurine, isoglutamine, choline, lactate, phenylalanine and tyrosine and decreased levels of lipids and triglycerides in tumour relative to adjacent healthy tissue, they cannot be used to determine the stage of the disease^88^ and may have limited application as biomarkers for early‐stage detection of the disease.

Proton nuclear magnetic resonance spectroscopy (^1^H NMR)‐based metabolomic approach in combination with pattern recognition through principal component analysis (PCA) and orthogonal partial least squares discriminant analysis (OPLS‐DA) was employed to profile faecal metabolites of CRC patients at different stages against healthy controls, HCs. OPLS‐DA revealed that each stage of CRC could be clearly distinguished from HC based on their metabolomics profiles. Relative to HCs, the CRC group depleted levels of acetate, butyrate, propionate, glucose, glutamine and increased quantities of succinate, proline, alanine, dimethylglycine, valine, glutamate, leucine, isoleucine and lactate were found in the CRC group. Of particular interest is that the faecal metabolic profiles of HCs can be distinguished from CRC patients, even in the early stage (stage I/II), emphasizing the potential utility of NMR‐based faecal metabolomics fingerprinting as predictors of earlier diagnosis in CRC patients. Additionally, glucose, lactate, SCFAs, glutamate and succinate at stage I/II significantly differed from those at stages III and IV.^81^ A later study by the same group investigated colonic tumour tissues and their normal adjacent tissues together with patient‐matched faecal samples obtained pre‐ and post‐operatively revealing the relationship between faecal metabolic phenotypes and changes in CRC metabolites. This analysis which also employed ^1^H NMR spectroscopy combined with pattern recognition technique showed that acetate compared to other identified metabolites was most profound in distinguishing the CRC group from healthy controls. Interestingly, faecal acetate correlated positively with changes of glucose and myo‐inositol in the tumour tissues indicating enhanced energy generation needed for rapid cell proliferation. Furthermore, significantly higher levels of lactate, glutamate, alanine, succinate, and reduced concentrations of butyrate, relative to the controls were found to be the overlapping discriminatory metabolites detected across sampled CRC tissues and faeces. They inferred that these metabolites may reflect tumour cell shedding and could also show metabolic pathway anomalies possibly revealing associations to enhanced glycolysis, tricarboxylic acid cycle, glutaminolysis and SCFA metabolism.^82^ This study, however, did not include adenoma patients, and this is essential and should be investigated be explored for future studies.

In the study of Clos‐Garcia, however, observable differences existed in faecal levels of sphingolipids and cholesteryl esters in CRC patients. Partial least squares discriminant analysis (PLS‐DA) was reported to differentiate the CRC groups from the adenoma/control groups but was not clearly distinct for the control and adenoma groups. The metabolites which mostly contributed to the distinction were the cholestryl esters and sphingomyelins. Glycerophosphatidylcholine (PC) species were also partly involved and PLS‐DA analysis in a pairwise manner clearly distinguished the CRC group from both the healthy control and adenoma groups.^40^ These researchers also showed that integrating metabolomics data with that of metagenomics was distinct in the separation of the healthy control group from CRC group as well as adenoma group from CRC group. More researches into these metabolites (sphingomyelins and cholestryl esters) in healthy, adenoma and CRC patients is recommended for the gut microbiota, faecal biomarker study. It is intriguing to note that the bacteria genera found to be differential in the three groups of individuals studied also correlated with the very same metabolite classes majorly observed to be discriminating and differential between sample groups.^40^

In furtherance, the development of models that integrate both metabolomics and metagenomics data of sampled individuals is strongly advocated for as such models may improve the sensitivity and specificity of detecting CRC at treatable stages which will in turn, prolong the life span of such individuals. There is no consensus yet on the applicability of the gut‐faecal microbiota metabolites as biomarkers for early detection of CRC (Table 2). However, some studies discussed above emphasize that it can be utilized. It is proposed that future studies should further investigate and validate these research findings especially in larger cohorts as the sample sizes utilized by some of the researchers were small.

**TABLE 2:** Summary of research findings on the application of metabolomics as biomarker.

Hypothesis	Technique	Metabolites	Major findings	Use as biomarker	References
Different intestinal diseases, such as AP and CRC could display particular SCFAs’ signature	GC‐MS	Acetic, butyric, propionic, isobutyric, isovaleric, valeric acids	The PLS‐DA model demonstrated a significant separation of CRC and AP groups from HC. AP showed higher levels of propionic acid and a lower level of isobutyric acid in comparison with CRC	Potential application in diagnosis of AP and CRC	^84^
Possible significant association between faecal SCFA concentrations and the presence of colonic adenomas or carcinomas	HPLC	Acetate, propionate, butyrate.	There were no significant associations existed between SCFA concentration and tumour status even with the random forest classification models.	Faecal SCFA concentrations have limited predictive power	^87^
How the gut bacteria composition impact host metabolism in the presence of adenoma and CRC could be assessed and deployed to identify potential non‐invasive, early biomarkers for disease	UHPLC‐MS metabolomics	Cholesteryl esters, sphingomyelins	Integration of metabolomics and microbiome data revealed tight interactions between bacteria and host and performed better than FOB test for CRC diagnosis	Identifies potential early biomarkers for CRC diagnosis	^40^
Analysis of the faecal metabolome (as a proxy of the gut metabolome) of adenoma patients, could be used to characterize biochemical signatures associated with the early events of CRC pathogenesis	UPLC‐MS/MS	Sphingolipids, secondary bile acids, polyunsaturated fatty acids	Bioactive lipids such as sphingolipids, secondary bile acids and polyunsaturated fatty acids were higher in adenoma compared to the controls and changes metabolites changes remained consistent in CRC patients	Observed changes in bioactive lipids were not significant enough to be employed as metabolic biomarkers in the early diagnosis of CRC	^80^
NMR‐based faecal metabolomics fingerprinting could be used as potential predictors of early diagnosis in patients with colorectal cancer	^1^H NMR	Glucose, lactate, SCFAs, glutamate, proline, succinate, isoleucine, leucine, valine, alanine, dimethylglycine, lactate	Faecal metabolic profiles of HCs can be well discriminated from those of early‐stage (stage I/II) CRC patients	Faecal metabolites can serve as early diagnostic biomarkers for CRC detection	^81^
The correlation between faecal metabolic phenotypes and those of tumour tissues could be utilized as biomarkers for early CRC detection	^1^H NMR	Lactate, glutamate, alanine, succinate, acetate	Faecal acetate correlated positively with changes of glucose and myo‐inositol in the tumour tissues	Acetate discriminated CRC group from HCs	^82^
Local colonic dysbiosis between tumour and normal mucosa may determine cancer‐stage and mucosal microbiome‐metabonome interactions	MAS‐NMR	Taurine, isoglutamine, choline, lactate, phenylalanine, tyrosine, lipids, triglycerides	16S rRNA gene sequencing revealed that microbiota ecology seems to be cancer stage‐specific and is strongly associated with histological features of poor prognosis	Limited application as biomarkers for early‐stage detection	^88^

Note

Abbreviations: ^1^H NMR, Proton nuclear magnetic resonance spectroscopy; AP, adenomatous polyposis; CRC, Colorectal cancer; GC‐MS, Gas chromatography‐mass spectrometry; GC‐MS, Gas chromatography‐mass spectrometry; HCs, healthy controls; HPLC, High‐performance liquid chromatography; MAS‐NMR, 1H Magic Angle Spinning Nuclear Magnetic Resonance spectroscopy; PLS‐DA, partial least squares discriminant analysis; SCFAs, Short‐chain fatty acids; UPLC‐MS/MS, Ultra‐performance liquid chromatography tandem mass spectrometry.

## 4 CHALLENGES AND FUTURE PERSPECTIVES

Studies on the gut microbiota and CRC are very important, provide deep insights into one of the possible mechanisms of the onset, progression and even prognosis of the disease. In essence, it reveals great potentials for its application in the early detection and treatment of CRC, particularly in personalised therapy. The intriguing facts about this research area are, however, not without challenges. Paramount among these challenges include the technicalities involved in sample collection and the type of sample collected. As stated previously,^15^ one major challenge facing this research area even though it makes a promising early detection strategy are the differences that tend to exist between mucosa‐associated microbiota and faecal microbiota. Although faecal samples are majorly employed, some studies have indicated that using tumour tissues from the gut of CRC patients may be more significant than faecal samples since microbial cells tend to adhere on the intestinal epithelium.^90^ Some studies that have employed this method reported that while *Fusobacterium*, *Parvimonas*, *Gemella* and *Leptotrichia* were the most significantly abundant bacteria in early‐stage CRC, fusobacteria and β‐*Proteobacteria* were more enriched as the cancer stage progressed.^65^, ^88^ Additionally, variations in sample collection, processing and method of data analysis applied by various studies also affect the gut microbiome‐faecal biomarkers study. Setting a standard procedure for these protocols will improve uniformity (reproducibility) and lead to more objective comparisons between various studies on the subject.

The cost‐effectiveness of any screening procedure is important for successful clinical application and affordability is one of the prominent requirements of a good biomarker. Some studies have stated that the application of faecal metagenomics in the diagnosis of CRC is affordable^38^; however, one notable factor that could contribute to increased diagnostic cost is usually the number of biomarkers employed by some of these studies which could range from 11 to 22. Many developing countries may not be able to integrate this method for CRC screening even though different studies have confirmed its sensitivity over many current non‐invasive screening tests.^91^ The most discriminatory screening markers should therefore be employed for CRC screening as its benefits will undoubtedly be far‐reaching spreading across populations of middle to low‐income source.

There is also the challenge of identifying additional markers with enhanced predictive value and eventually validating them in much larger cohorts involving different nationalities. The prime focus of such exploration would be to discover faecal metagenomics markers that have strong predictive power in diagnosing early‐stage CRC, leading to significantly reduced CRC‐associated mortality.^38^ The data presented in Table 1 show that various studies have tried to validate faecal microbiota signatures in different populations. However, the development of a few defined universal/regional‐based faecal microbiota panels is essential in order to reduce cost and to overcome the hurdle posed by various factors that could affect the diversity and composition of the gut microbiota. While this is being carried out, it is important to note that some studies have also shown that the *Fusobacterium* genera alone cannot be used in the distinction between adenoma and carcinoma patients as there is no significant association between this bacterium and the location or stage of the carcinomas.^40^, ^69^, ^71^ It is reported to give better predictions on CRC, particularly in the advanced stage but less accurate at the early stage.^73^ Future studies can therefore explore the best microbial biomarker panels that integrate *Fusobacterium* spp. for early‐stage diagnosis of CRC.

The genera *Adlercreutzia* should be further investigated in diverse races and ethnic groups to validate its prospect as an efficient diagnostic biomarker of adenoma. Studies on equol production by these bacteria is needed taking the dietary patterns of participants in consideration, and the effect of *Adlercreutzia* in adenoma patients should be further studied since the equol it produces is associated with a lower risk of CRC.^40^

It is important to mention that although many studies have combined the faecal microbial biomarkers identification with FIT in the early detection of CRC,^13^, ^18^, ^70^, ^91^, ^92^, ^93^ one major challenge that has always been encountered is the increase in the number of false‐positive results. The sensitivity obtained by either of these methods is almost always enhanced when they are both combined into a single model for adenoma detection. However, a corresponding improvement in specificity is usually not observed and is rather, reduced. This makes these combination models still not very suitable for early CRC detection as the goal is to reduce the number of primary diagnoses by colonoscopy. Hence, further studies on FIT‐faecal microbiota panel combination models are strongly advocated for and even other models that can explore faecal microbial signatures with other non‐invasive screening biomarkers for early CRC detection is encouraged.

**FIGURE 3:**
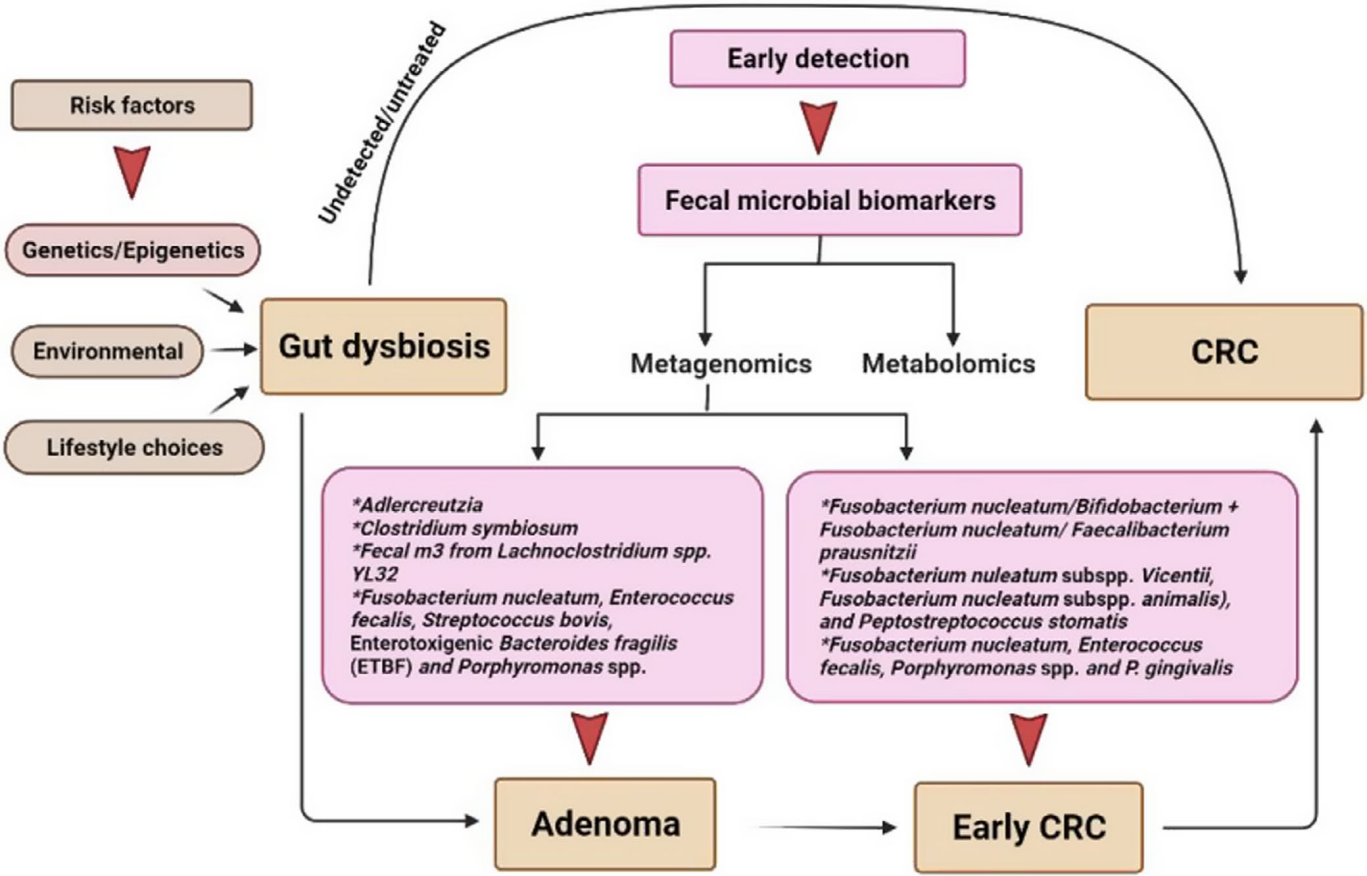
Detecting CRC at the early stage increases chances of survival and colonoscopy, the gold standard for CRC diagnosis is both expensive and invasive. At present, sensitive, specific, cost‐effective and non‐invasive methods are urgently needed. CRC‐related gut dysbiosis is implicated in the onset and progression of the disease and specific gut microbes found in faecal samples of patients could serve as biomarkers

A graphical presentation summarizing the data and ideas discussed in the manuscript is illustrated in Figure 3.

## 5 CONCLUSION

The gut microbiota has been shown to provide a reservoir of biomarkers for the early diagnosis of CRC. Early detection of CRC would grossly reduce the high mortality associated with CRC. The best faecal biomarker panel that will improve both the sensitivity and specificity of adenomas detection either applied alone or even in combination with other non‐invasive CRC diagnostic methods is achievable and can also be made affordable. This will ensure that more persons are screened and more lives, saved.

## CONFLICT OF INTEREST

The authors declare that there are no conflicts of interest.

## AUTHOR CONTRIBUTION

**Chinasa Valerie Olovo:** Conceptualization (equal); Data curation (equal); Investigation (equal); Methodology (equal); Project administration (equal); Resources (equal); Software (equal); Writing‐original draft (equal). **Xinxiang Huang:** Conceptualization (equal); Data curation (equal); Formal analysis (equal); Investigation (equal); Methodology (equal); Project administration (equal); Resources (equal); Validation (equal); Writing‐original draft (equal). **Xueming Zheng:** Formal analysis (equal); Methodology (equal); Resources (equal); Validation (equal); Writing‐review & editing (equal). **Min Xu:** Conceptualization (equal); Funding acquisition (equal); Project administration (equal); Supervision (equal); Validation (equal).
